# The Use of Non-i nvasive Biomarkers to Screen for Advanced Fibrosis Associated with Metabolic Dysfunction-associated Steatotic Liver Disease in People with Type 2 Diabetes: A Narrative Review

**DOI:** 10.17925/EE.2025.21.1.4

**Published:** 2025-02-20

**Authors:** David M Williams, Jagadish Nagaraj, Jeffrey W Stephens, Thinzar Min

**Affiliations:** 1. Department of Diabetes and Endocrinology, Morriston Hospital, Swansea, UK; 2. Department of Hepatology and Gastroenterology, Morriston Hospital, Swansea, UK; 3. Diabetes Research Group, Swansea University Medical School, Swansea University, Swansea, UK; 4. Department of Diabetes and Endocrinology, Neath Port Talbot Hospital, Swansea, UK

**Keywords:** Liver disease, liver fibrosis, metabolic dysfunction-associated steatotic liver disease, metabolic syndrome, screening, steatohepatitis, type 2 diabetes

## Abstract

There is growing interest in metabolic dysfunction-associated steatotic liver disease (MASLD), given its increasing prevalence and our developing understanding of the disease. People living with type 2 diabetes or obesity have a greater risk of developing significant hepatic steatosis and a greater risk of more rapid progression to steatohepatitis, advanced hepatic fibrosis and hepatocellular carcinoma. As such, various international bodies now advocate for routine screening for MASLD-related hepatic fibrosis in people with such risk factors. This would permit earlier targeted lifestyle interventions and the use of pharmacotherapies, which may reverse earlier stages of MASLD-associated fibrosis. This may improve both liver-related and cardiovascular outcomes in these higher-risk groups. Nonetheless, the identification of MASLD-related hepatic fibrosis is frequently limited to liver enzyme tests, given the lack of a systematic approach to investigation and screening. In this article, we discuss the potential to screen for advanced fibrosis in people with MASLD using various blood-based biomarkers, such as the Fibrosis-4 score, non-alcoholic fatty liver disease fibrosis score and enhanced liver fibrosis test, amongst other available patented and non-patented tests. We discuss the relative benefits and limitations of each and the potential for future research in this evolving area of clinical interest.

## Article highlights

- There is growing clinical importance attributed to the development of metabolic dysfunction-associated steatotic liver disease in people with type 2 diabetes (T2D).
- Numerous international groups now advocate screening for advanced fibrosis in people with risk factors, such as T2D, using non-i nvasive biomarkers.
- This article explores the rationale to screen for advanced fibrosis in people with risk factors and the potential screening processes.
- Patented and non-patented blood-based biomarker tests for advanced fibrosis are reviewed, including the original validation studies and limitations in practice.
- Areas of interest in future research are discussed, and considerations for better evidence-based implementation in practice and the need for new biomarkers are highlighted.

Metabolic dysfunction-associated steatotic liver disease (MASLD) is the most common form of liver disease, affecting around a third of the global population.^1,2^ There is growing interest in MASLD due to the major global prevalence of the disease and the potential scale of associated complications, including those of decompensated liver disease, cardiovascular events and malignancies amongst others.^3^ This, amongst other concerns, prompted a recent change in the nomenclature and criteria used to diagnose and classify the disease.^4^ Given the recent advances in trial outcomes seen with pharmacological therapies for MASLD, which were previously lacking, there are now mounting calls to screen for MASLD-related advanced hepatic fibrosis to guide therapies to prevent disease progression and associated complications.^5,6^

People living with features of the metabolic syndrome, such as type 2 diabetes (T2D), obesity, hypertension and/or dyslipidaemia, are at greater risk of MASLD, and MASLD is often seen as an extended feature of the metabolic syndrome. People living with T2D have a twofold greater prevalence of MASLD, affecting two-thirds of people with T2D globally.^7^ However, these associations are increasingly complicated, involving shared genetic factors, the gut microbiome, bile acid dysregulation and environmental factors that accelerate disease progression.^8,9^ Ultimately, people with T2D are both more likely to have MASLD and are more prone to developing MASLD-related advanced fibrosis with cardiovascular issues than those without T2D, frequently resulting in poorer clinical outcomes.^10^

The difficulty in identifying MASLD is twofold. First, diagnosis of MASLD continues to often rely upon invasive testing with liver biopsy, though there is growing confidence around the use of some non-i nvasive tests to identify the disease.^11–14^ This is supported by recent changes in disease definition and classification, which give clinicians better assurance to make a diagnosis based on non-i nvasive tests alone.^4^ Nevertheless, liver biopsy continues to be needed in some, such as those with competing aetiologies for chronic liver disease (CLD), unclear degree of fibrosis or for inclusion in clinical trials.^14^ Second, given that MASLD is a dynamic spectrum of disease including hepatic steatosis, metabolic dysfunction-associated steatohepatitis, liver cirrhosis and hepatocellular carcinoma, the frequency of screening is difficult to establish. This is more complicated in people living with T2D and/or obesity, as these co-morbid disease states result in a precipitous progression to advanced fibrosis, with a greater degree of disease heterogeneity.^15^ Still, many people with MASLD-related cirrhosis may be asymptomatic for years and remain unidentified with a risk of poorer health outcomes, including all-cause mortality, cardiovascular disease, decompensated liver disease, hepatocellular carcinoma and other malignancies that increase in tandem with hepatic fibrosis.^3^ Beyond the personal and often concealed health burden of MASLD-related fibrosis, there is a growing economic burden. It is estimated that the excess healthcare spent on patients with MASLD is around twice that of age-matched controls, and this continues to increase with increasing stages of fibrosis. Moreover, in the USA, Europe and Hong Kong, at least, these healthcare costs are expected to continue to rise.^16^

Given the rising prevalence of MASLD and its risk factors, with growing recognition of both the personal and economic health costs, there are calls to screen for MASLD earlier in the natural course of the disease. Screening for MASLD and associated fibrosis in people at greater risk, such as those living with T2D, is an appealing model given the comparative health and cost benefits versus general population screening. Moreover, this may support the use of specific pharmacological measures to prevent progressive fibrosis and permit earlier specialist and multidisciplinary management. Even so, there remains debate around the plausibility of MASLD screening, even in people with additional risk factors. There also remain uncertainties around the tests used, the economic impact of MASLD screening and some debate around the additional therapeutic interventions that might be undertaken to reverse MASLD-related fibrosis if identified in people with T2D.

## Rationale for MASLD screening in people with type 2 diabetes

For more than 50 years, there have been established broad principles that are required to be met when considering the cogency of developing a new screening programme, initially proposed by Wilson and Jungner in 1968.^17^ These principles are summarized in *Table 1*.^17^ Given the major progress over the last 10–20 years in our understanding of MASLD, which has more recently helped to develop disease-specific treatments, most of these principles to support the implementation of a screening programme are satisfied with respect to identifying MASLD in people with T2D.

### MASLD is an important disease that is increasingly understood

First, as a highly prevalent global disease state associated with poor outcomes, MASLD-related fibrosis is clearly an important health problem.^3^ However, simple hepatic steatosis itself does not strongly associate with important health outcomes in the absence of significant fibrosis, so the specific disease state that should be screened for is advanced hepatic fibrosis in association with MASLD.^3,11–14,18^ However, if excess hepatic fat is identified, it is still an important outcome measure as it may indicate those at greater risk of progressive steatohepatitis or fibrosis, and this finding alone can motivate the affected person to improve diet and lifestyle efforts in people with T2D, at least, to reduce their risk of progressive liver disease.^19^ It is increasingly understood that multiple factors act synergistically to induce hepatic steatosis and steatohepatitis.^8,9^ These include factors that affect the supply and metabolism of intrahepatic lipids. First, the supply of intrahepatic lipids is closely associated with excess energy intake, especially dietary sugars and saturated fats, which drive *de novo* lipogenesis.^20,21^ Second, normal lipid metabolism in both hepatic and adipose tissue is disrupted by insulin resistance to induce greater lipolysis and free fatty acid release, whilst various exercise regimes can improve both lipid metabolism and liver health mutually.^22,23^ Additionally, the gut microbiome, bile acid dysregulation and genetic factors can further influence the degree of hepatic fat deposition and risk of progressive steatohepatitis.^8,9^ Over time, these factors cause progressive steatohepatitis, eventually resulting in significant hepatic fibrosis.

### MASLD is often a reversible disease state

Earlier stages of steatosis or steatohepatitis are more likely reversible, and diet and lifestyle interventions continue to be seen as the cornerstone of management.^11,13,14^ Lifestyle interventions are well established to associate with important reductions in measures of steatohepatitis and reduced fibrosis progression, in addition to well-established general health benefits.^24–27^

**Table 1:** The Wilson and Jungner principles of any screening programme^17^

Suggested requirements for the basis of any screening programme
(i) The condition(s) represent an important health problem
(ii) The natural history of the condition(s) is well understood
(iii) There are recognizable latent and early stages of these conditions
(iv) There are appropriate tests with acceptable accuracy, sensitivity and specificity
(v) The test is acceptable to the screening population
(vi) It should be clear on whom to treat as patients
(vii) There should be acceptable treatment available for those identified with the disease
(viii) Any facilities required to provide further diagnostic tests and treatment should be available to the screening population
(ix) The cost of screening, further investigations and treatment of patients with the identified conditions(s) should be economically balanced
(x) Finding people with the condition(s) should be a continuing process

*The original principles of any screening programme proposed by Wilson and Junger (1968).^17^*

Drug therapies have been shown to improve both steatosis and steatohepatitis for more than a decade, including pioglitazone, vitamin E, semaglutide and tirzepatide.^6,28,29^ However, it was not until 2024 that pharmacotherapies were associated with statistically significant reductions in MASLD-associated fibrosis; now, resmetirom has been shown to do so in a prospective trial.^5^ Therefore, it is likely that this drug could be used for the specific treatment of significant (e.g. stage Ib-3) hepatic fibrosis associated with MASLD, and resmetirom is already approved for use by the Food and Drug Administration in the USA.^30^ Moreover, numerous agents are in development for the treatment of MASLD and early stages of MASLD fibrosis. These include repurposed therapies traditionally used to treat T2D or obesity, as well as liver-specific agents, which have been recently reviewed elsewhere.^31^ Thus, identifying people with MASLD at earlier fibrosis stages could lead to targeted therapeutic intervention to reverse the disease. However, further prospective interventional trials to evaluate the impact of novel drug therapies on the risk of major adverse liver outcomes (MALO), such as decompensated liver disease, hepatocellular carcinoma and cardiovascular outcomes, are needed.^32^ Nonetheless, previous observational studies using Swedish healthcare registers exploring the risk of MALOs associated with the use of glucagon-l ike peptide-1 receptor agonists in people with T2D recently showed therapeutic promise in this area.^33^ Moreover, there is growing discussion about the use of bariatric surgery as a tool in the treatment of MASLD-related fibrosis or even hepatic cirrhosis, as MASLD-related fibrosis is increasingly recognized as an obesity-related comorbidity that could be treated with metabolic surgery.^13,34^ These treatments are best considered and delivered by a multidisciplinary team, including hepatologists, endocrinologists, primary care physicians, specialist nurses and dieticians, amongst others.^14^

### Barriers to screening for MASLD

Nevertheless, there remain important barriers to implement screening for advanced MASLD-related fibrosis in people with T2D. Given the high population prevalence of T2D, the screening programme strategy would need the resources not only to regularly deliver the screening test but also to review and treat those identified at risk of advanced MASLD-related fibrosis. Within this high-risk population, there is potential for the referral burden to overwhelm services, given the estimated global prevalence of clinically significant and advanced fibrosis in people with T2D and MASLD is 40.8 and 15.5%, respectively.^7^ However, this also highlights the scale of the untreated population that could benefit from improved investigation and clinical management. In view of increasingly precious healthcare resources, this is a consideration that requires careful evaluation. Current evidence suggests that some screening strategies in either high-risk or community-based populations may be cost effective.^35–38^ Indeed, in Thai patients with metabolic syndrome, ultrasound was cost effective to screen for MASLD, whilst screening with transient elastography (TE) or the Fibrosis-4 (FIB-4) score was cost effective in American, Singaporean and British cohorts.^39–42^ However, other analyses indicate that screening for steatohepatitis in people with T2D may not be cost effective.^38,43^ Further studies are needed using specific screening algorithms that are feasible to deliver (e.g. blood-based algorithms) in practice. For example, initial screening with TE is unlikely to be practical on a national basis due to the training and staff time involved in delivering this, notwithstanding the initial cost of the devices needed, whilst liver biopsy is not appropriate for screening in view of the associated risks. Moreover, updated cost analyses that can include the health benefits and cost of newer medicines to reverse hepatic fibrosis would be of major interest and importance before screening can be introduced into routine practice.

Like other complications associated with T2D, supporting patients to attend for regular screening is important, though may be challenging. This is because MASLD is typically asymptomatic for long periods, and patients with T2D may not be aware of MASLD or its significance.^44–46^ Moreover, people living with T2D already undergo an extensive annual review for complications, and further tests or appointments may prompt patient disengagement. Screening tests using blood-based biomarkers within pre-existing primary and secondary care programmes would therefore be most practical to minimize additional appointments and investigations. Moreover, including discussion about MASLD in diabetes education programmes for patients may support their understanding of MASLD and its relevance to their health, like microvascular and macrovascular complications.

**Figure 1:**
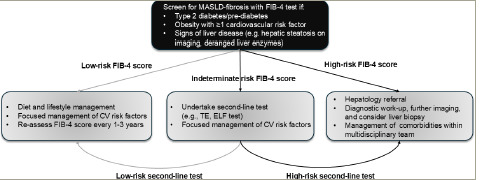
Proposed screening approach for MASLD-related fibrosis^11,13,14^

Given the high prevalence of T2D, the screening strategy would need to be deliverable on a large scale. Following a primary screening test using a blood-based biomarker, those deemed ‘at risk’ of advanced fibrosis could move to a second-l ine test to confirm the need for hepatology referral. Naturally, such pathways would need to be deliverable at both national and local levels. Indeed, screening strategies adopting this general approach have been proposed in recent years and are currently supported by both European and American hepatology and diabetes international advisory groups.^11,13,14^ These groups suggest a broadly similar approach, with the use of the FIB-4 score as the initial screening test in those with risk factors or signs of disease (e.g. T2D, obesity, cardiovascular risk factors, deranged liver enzymes or hepatic steatosis on abdominal imaging). For people with a FIB-4 score ≥1.30 (≥2.0 if aged >65 years), a second-l ine test, such as TE or the enhanced liver fibrosis (ELF) test, should be used. Referral to hepatology services is recommended if these tests support moderate to advanced fibrosis. For people with a low FIB-4 score, it should be considered to intensify the management of their comorbidities and cardiovascular risk factors, with a view to repeat the test every 1–3 years. A schematic summarizing this screening approach is presented in *Figure 1*. Interestingly, studies undertaking additional screening tests for liver fibrosis in high-risk individuals show that this can support patients to make changes in their lifestyle to reduce the risk of either alcohol-related liver disease (ALD) or MASLD fibrosis.^19^

There are benefits and limitations for each of the blood-based biomarkers studied to date to screen for MASLD-related fibrosis, which are discussed below.

*A general schematic summarizing the proposed screening approach for MASLD-related fibrosis by the American Association of Clinical Endocrinology, American Association for the Study of Liver Diseases, European Association for the Study of the Liver, European Association for the Study of Diabetes, and European Association for the Study of Obesity. CV = cardiovascular; ELF = enhanced liver fibrosis test; FIB-4 = Fibrosis-4 score; MASLD = metabolic dysfunction-associated steatotic liver disease; TE = transient elastography.*

**Table 2:** Currently available blood-based tests to identify advanced hepatic fibrosis^49–59^

Biomarker test	Components	Original validation	Benefits	Limitations
**Non-patented**				
FIB-4 score	Age, ALT, AST and platelet count	People with HCV^49^	Easily available, cheap, unaffected by BMI, good NPV and predicts outcomes	Performs poorly in those aged <35 or >65 years
NFS	Age, albumin, ALT, AST, BMI, IFG/T2D status and platelet count	People with MASLD^50^	Easily available, cheap and predicts outcomes	Less accurate in people with obesity, T2D and age <35 or >65 years
APRI	AST and platelet count	People with HCV^51^	Easily available and cheap	Modest accuracy compared with other tools
BARD score	BMI, AST:ALT ratio and T2D status	People with MASLD^52^	Easily available and cheap	Limited PPV and concern over BMI threshold in some ethnic groups
**Patented**				
ELF test	HA, PIIINP and TIMP-1	Various causes of CLD^53,54^	Good accuracy and predicts outcomes	Cost, some unavailability, FP in connective tissue disorders and influenced by age/gender
ADAPT score	Age, platelet count, presence of T2D and PRO-C3 level	People with MASLD^55^	Good accuracy and useful in people with obesity	Cost and limited availability
FibroMeter-NAFLD	Age, ALT, AST, body weight, ferritin, glucose and platelet count	People with MASLD^56^	Good accuracy	Cost, limited availability, less accurate than FibroMeter^V2G^^57^
FibroTest	α2 macroglobulin, age, apolipoprotein-A1, bilirubin, gender, GGT and haptoglobin	People with HCV^58^	Good accuracy and associates with liver-related outcomes	Cost, limited availability, affected by Gilbert’s, haemolytic and inflammatory conditions
Hepascore	α2 macroglobulin, age, bilirubin, gender, GGT and HA	People with HCV^59^	Good accuracy and predicts allcause and liver-related deaths	Cost, limited availability, affected by Gilbert’s, haemolytic and inflammatory conditions

*A summary of the available patented and non-patented non-invasive blood-based biomarkers used to determine the risk of advanced fibrosis.*

*ADAPT = age, presence of diabetes, PRO-C3 and platelet count; ALT = alanine transaminase; APRI = AST-to-platelet ratio index; AST = aspartate aminotransferase; BARD = BMI-AST:ALT ratio-diabetes mellitus; BMI = body mass index; CLD = chronic liver disease; ELF = enhanced liver fibrosis test; FIB-4 = Fibrosis-4 score; FP = false positive; GGT = gammaglutamyl transferase; HA = hyaluronic acid; HCV = hepatitis C virus; IFG = impaired fasting glucose; MASLD = metabolic dysfunction-associated steatotic liver disease; NAFLD = non-alcoholic fatty liver disease; NFS = NAFLD fibrosis score; NPV = negative predictive value; PIIINP = aminoterminal propeptide of type 3 procollagen; PPV = positive predictive value; PRO-C3 = N-terminal pro-peptide of type 3 collagen; T2D = type 2 diabetes; TIMP-1 = tissue inhibitor of matrix metalloproteinase-1.*

## Biomarkers available for MASLD fibrosis screening

Given the poor correlation between liver enzymes such as alanine transaminase (ALT) or aspartate aminotransferase (AST) with the degree of hepatic fibrosis in MASLD and other liver diseases, numerous prediction scores have been developed using a combination of biochemical values and anthropometric data to better predict fibrosis.^47^ Some of these tests were developed in cohorts with other disease states, such as viral hepatitis or ALD, and the interpretation of the results from each test may not be the same across different aetiologies of CLD.^48^ These tests include the ADAPT (age, presence of diabetes, PRO-C3 and platelet count) score, APRI (AST-to-platelet ratio index), AST:ALT ratio, BARD (body mass index [BMI]-AST:ALT ratio-diabetes mellitus) score, ELF test, FIB-4 score, FIBROSpect II, FibroTest, FibroMeter and the non-alcoholic fatty liver disease (NAFLD) fibrosis score (NFS), amongst others. The variables used in each of these algorithms are shown in *Table 2*, and each of these scores is discussed further below.^49–59^ Simple fibrosis scores that use routinely collected clinical data, such as simple biochemical tests and anthropometric data, are preferred due to their relative ease of use and lower cost. However, there are an increasing number of advantageous patented risk scores available for use in people with MASLD.

### Non-patented fibrosis scores

#### The FIB-4 score

The most established of these is the FIB-4 score, which uses the patient’s age, ALT, AST and platelet count to establish a risk score. Initially developed to determine the risk of advanced hepatic fibrosis in people living with hepatitis C virus (HCV) infection, it has been since validated in people with MASLD.^49,60^ Those with a score <1.30 have low risk, those with a score 1.3–2.67 have intermediate risk and those with a score >2.67 have a high risk of advanced fibrosis. However, in those aged <35 or >65 years, the accuracy of the score to exclude advanced fibrosis is diminished, meaning some suggest that a score of 2.0 is needed in people aged >65 years.^61,62^ Given the good accuracy and potential for use in the initial stages of a sequential screening algorithm with other tests, there is much interest in this test.^63^ Moreover, the test has excellent negative predictive value (NPV), is easily available without excess cost, is relatively reliable in people living with obesity, can predict both liver-related morbidity and mortality and is a useful biomarker in the monitoring of fibrosis progression in people with MASLD, making it is an attractive biomarker for advanced fibrosis in MASLD.^64–69^ Given these factors, this is the preferred initial screening test suggested by current American and European guidance in people with MASLD risk factors such as T2D or obesity.^11,13,14^

#### The NFS

The NFS is another widely used simple fibrosis score tool, as it includes the routinely collected variables of age, albumin, ALT, AST, BMI, platelet count and the presence of impaired fasting glucose or T2D. This score was also primarily derived from a cohort of people living with MASLD.^50^ Herein, a score less than -1.455 has an excellent NPV for advanced fibrosis, whilst a score between -1.455 and 0.676 implies intermediate risk, and >0.676 suggests a high risk of advanced fibrosis. However, the NFS is not usually recommended in the screening of MASLD-related fibrosis in high-risk cohorts by recent American and European advisory groups because it has poorer accuracy and clinical utility to screen for advanced fibrosis in at-risk groups including people living with T2D, obesity and in those aged <35 years or >65 years.^11,13,14,62,64,70^ Although it may still provide some useful information regarding fibrosis progression, liver-related morbidity and mortality in people with MASLD, it may be useful as part of a sequential test algorithm but probably less effective than the FIB-4 score.^63,65–67^

#### The APRI

The APRI is another clinically useful scoring tool to screen for advanced fibrosis, as it again uses routinely collected clinical variables of AST and platelet count. Like the FIB-4 score, the APRI was originally developed in people living with HCV infection and was later also validated in people with MASLD.^51,71^ There are mixed observations with respect to its accuracy. Although it is generally considered to be less accurate than the FIB-4 score and NFS, it is not recommended in current guidance to screen for MASLD-related advanced fibrosis.^11,13,14,67,72,73^ This may be due to other factors that significantly influence the AST or platelet count, which can have a greater impact on this value, as only these two values are used to generate the score. Nonetheless, the score is useful in monitoring progressive fibrosis, predicting adverse liver-related outcomes and is associated with the occurrence of hepatocellular carcinoma in people with MASLD.^65,67,74^

#### The BARD score

The BARD score was developed as another simple scoring tool to predict advanced fibrosis, originally in a cohort of people with MASLD.^52^ A discrete numerical score of 0–4 is derived by giving one point for individuals with BMI≥28.0 kg/m^2^, two points if the AST:ALT ratio is >0.8 and a further point if there is a history of T2D. A score of 0–1 had an excellent NPV of 96% in people with MASLD under tertiary care follow-up.^68^ However, there are concerns about using the BMI cut-off of ≥28.0 kg/m^2^, particularly regarding its applicability to some ethnic groups, such as people of South Asian descent, who frequently develop MASLD at a lower BMI than other ethnic groups. Additionally, in the context of focussed screening (as opposed to population screening) in those with risk factors such as T2D or obesity, all would have a score ≥1, limiting its value in excluding disease. The score is also less accurate in predicting advanced fibrosis, risk of all-cause mortality and hepatic decompensation than other simple risk scores such as the FIB-4 score and NFS.^66,72,73,75^ This score is an expansion on the previous use of the AST:ALT ratio in the investigation of hepatic fibrosis. The AST:ALT ratio was originally developed in 1957 to help identify the cause of hepatitis, in which a ratio ≥2.0 suggests ALD, and a reduced ratio indicates viral hepatitis.^76^ In patients with MASLD, the AST:ALT ratio is typically <0.8, reflecting a greater rise in ALT than AST. However, as MASLD fibrosis progresses, the AST:ALT ratio rises, limiting its use in distinguishing the cause of fibrosis.^77^ The AST:ALT ratio alone has an excellent NPV ≥90%, but its poor positive predictive value limits its use in screening.^68^ Neither the BARD score nor the AST:ALT ratio are recommended for screening people at risk because of these important limitations.^11,13,14^

### Patented fibrosis scores

#### The ELF test

Given some of the limitations associated with the use of the simple risk scores above, more specific biomarker panels have been developed to improve diagnostic accuracy. The most validated of these is the ELF test, which currently comprises three serum biomarkers: aminoterminal propeptide of type 3 procollagen, hyaluronic acid (HA) and tissue inhibitor of matrix metalloproteinase-1. However, variables included within the ELF test algorithm have changed from its initial validation in people living with CLD from a range of aetiologies, having been subsequently simplified and validated with excellent accuracy for advanced fibrosis in people with MASLD.^53,54^ Whilst most original validation studies suggested a cut-off of 9.8 to evaluate for advanced fibrosis, in the UK, the National Institute for Health and Care Excellence recommends a higher score of 10.51, as a test threshold based largely upon cost analyses.^54,78,79^ Despite the accepted greater accuracy and increasing availability of this patented test, it can be adversely influenced by gender, advancing age and coexisting disease states with higher collagen turnover or inflammatory state, thereby generating false positive results.^78,80–82^ However, this is likely less of a concern compared with other non-i nvasive biomarker test tools, and the ELF test correlates well with liver-related and other major health outcomes either in people with suspected liver disease or in the general population.^78,83,84^ Given these benefits, the ELF test is frequently the preferred test for evaluating advanced MASLD-related fibrosis, according to recommendations from various hepatology and diabetes international groups. However, it is usually used as a second-l ine test following the FIB-4 score due to its relative cost and accessibility.^11,13,14,79^

#### The ADAPT score and PRO-C3

Another biomarker identified for the identification of advanced fibrosis in MASLD is the N-terminal pro-peptide of type 3 collagen (PRO-C3), which is a collagen fragment released in the process of developing (hepatic) fibrosis. This has good accuracy in detecting advanced fibrosis in people with MASLD even when used alone, but its clinical utility is improved by its inclusion within the ADAPT score.^55,85–87^ The ADAPT score includes age, platelet count, the presence of T2D and the PRO-C3 level, originally developed in a cohort of people with MASLD.^55^ Whilst there are a relatively limited number of studies exploring this score compared with other risk scores such as the ELF test or FIB-4 score, it has outperformed several non-patented scores such as the FIB-4 score, NFS, APRI and BARD previously.^55,86,87^ Indeed, one study observed that both the ADAPT and FIB-4 scores were the most accurate of numerous non-i nvasive measures of advanced (≥F3) or clinically significant (≥F2) fibrosis in people living with obesity.^64^ Nonetheless, this biomarker is not routinely available and is relatively expensive, which limits its use in current clinical practice.

#### The FibroMeter

FibroMeter is a series of various patented assays, each predicting the risk of advanced fibrosis in a range of different CLD aetiologies. The FibroMeter-NAFLD score comprises an algorithm including age, ALT, AST, body weight, ferritin, glucose and platelet count, previously shown to be useful in the detection of clinically significant fibrosis (≥F2), but has similar accuracy as other risk-predictive scores for detection of advanced fibrosis and cirrhosis.^56,88,89^ Subsequently, the FibroMeter Virus second-generation (FibroMeter^V2G^) test, which was originally developed for use in people with HCV infection, was found to be more accurate for advanced fibrosis than FibroMeter-NAFLD in people with MASLD by one meta-analysis and in a head-to-head study.^57,71^ Indeed, the Fibrometer^V2G^ has similar accuracy for advanced fibrosis as the ELF test in people with MASLD and was associated more significantly than other non-i nvasive tests with all-cause mortality and extra-hepatic mortality in one study.^71,90^ However, these tests have significant costs and are not universally available, limiting their recommendation in current international guidance and clinical utility. However, they may be a useful second-l ine non-i nvasive test in combination with TE in some circumstances to avoid the need for liver biopsy.^91^

#### The FibroTest

The FibroTest was initially developed for use in people living with HCV infection and was observed to have a 100% NPV and a good positive predictive value in the original validation study.^58^ It was subsequently shown to have good accuracy for the detection of advanced fibrosis in a subsequent study including people with MASLD.^92^ The score comprises α2 macroglobulin, age, apolipoprotein A1, bilirubin, gamma-glutamyl transferase (GGT), gender and haptoglobin. Later studies observed a strong association between the FibroTest score and liver-related death in people with MASLD or other forms of CLD irrespective of the patients’ BMI or T2D status.^93^ However, the FibroTest result can be confounded by non-MASLD-related factors, including Gilbert’s syndrome, haemolytic disorders or inflammatory conditions that may lead to false positive results.^94^ Moreover, the test remains relatively expensive with limited international availability compared with the other non-i nvasive biomarkers, which hinders its use in routine clinical practice, and much of the evidence on the use of this test is derived from people living with viral hepatitis, again limiting the use in people with risk factors for MASLD such as T2D or obesity.

#### The Hepascore

The Hepascore uses α2 macroglobulin, age, bilirubin, gender, GGT and HA to predict the risk of advanced fibrosis. Originally, this score was also validated in people living with HCV infection.^59^ In one head-to-head study comparing several non-i nvasive tests for advanced fibrosis in people with HCV, including the APRI, FibroMeter and FibroTest, the Hepascore demonstrated at least comparable accuracy.^95^ In a similar comparative study of non-i nvasive blood-based biomarkers in people with MASLD, the Hepascore was one of the most accurate tests to predict advanced fibrosis, compared with the APRI, BARD score, FIB-4 score, FibroMeter-NAFLD, FibroMeter^V2G^, FibroTest and NFS, and was also an accurate predictor of all-cause, liver-related and extra-hepatic mortality in this group.^71^ However, this biomarker panel remains both relatively unavailable and more expensive compared with other tests, especially compared with the simple fibrosis markers that limit its clinical use.

## Considerations for future research

There have been major advances in both the diagnostic tests and therapies available for people with MASLD and MASLD-related fibrosis over the last 20–30 years, alongside major progress in the clinical care of people living with other forms of CLD. Moving forward, future work should look to translate some of these lab or trial-based observations into practice for MASLD, and current research priorities have been highlighted by a recent Delphi consensus.^96^ Initially, this may be to develop or validate an appropriate screening approach, expanding upon recently recommended strategies by various international groups in adults with risk factors such as T2D or obesity, at least.^11,13,14^ Whilst some well-designed prospective studies have shown promise and highlighted the benefits of undertaking screening in at-risk populations, these studies have applied variable non-invasive tests to different cohorts, including populations under primary or secondary care with differing study inclusion criteria.^42,97,98^ Moreover, these studies were generally undertaken in European and US cohorts, and the application of such tests in other populations is needed. This may be of particular interest in children and adolescents, given the rising incidence of MASLD in younger people, with an estimated MASLD prevalence of 7–14%.^99^ There is a weaker evidence base validating the use of these non-i nvasive biomarkers in this group, although there has been some recent progress in this area of interest.^100,101^ Beyond the prospective cross-sectional studies to date, longer-term prospective studies to explore the appropriate frequency of screening in different population groups would be of interest. For example, to investigate whether the frequency of MASLD screening in people with T2D should vary in those with relatively poor glycaemic control or living with obesity, which are known risk factors to confer greater disease heterogeneity.

Despite our increasing understanding of MASLD and the importance of genetic factors and the gut microbiome on its development and progression, these factors currently have little impact in practice. However, genetic testing and specific treatments may be used in those presenting with signs of specific causes of steatohepatitis, such as coeliac disease, hypobetalipoproteinaemia, nutrient deficiencies, Wilson’s disease or other inherited metabolic diseases.^13,14,22^ However, recent studies highlight the potential use of the gut microbiome to identify MASLD in the broader population. Gut microbiome patterns identified MASLD with good accuracy using machine learning algorithms in one recent study, and preclinical studies highlight that modulation of gut bacteria using probiotics, antimicrobials or genetically modified bacteria lessens MASLD progression.^102–104^ This may be a result of their impact on host epigenetics, reviewed elsewhere.^104^ This is a fascinating area of active research, given the potential for personalized therapies. Nonetheless, clinical trials to evaluate this therapeutic approach are needed.^13,14,22^

The major contention around MASLD screening is based on the cost effectiveness of various approaches, although many of the cost analyses to date have observed that screening in various settings is likely to be cost effective.^35–38^ However, as these studies have been taken in various populations (some of which are hypothetical cohorts) and apply different non-i nvasive tests in populations with differing risks of MASLD fibrosis, there remains some caution with screening implementation. However, one UK-based cost analysis derived from a prospective study based in primary care found that the use of the ELF test, FIB-4 score or TE to screen for advanced fibrosis in MASLD is likely cost effective.^42^ Cost analyses of other prospective studies in different clinical settings or in different populations would be of major interest. Such cost analyses may be improved by updated cost–benefit data associated with the use of resmetirom, given its impact on fibrosis. However, they will continue to be limited by the absence of prospective data on their impact on MALOs and major adverse cardiac events (MACE). It remains to be proven that reversal of MASLD-related fibrosis reverses the associated complications seen in people with progressive fibrosis, although this may be expected given the impact of this drug on numerous risk factors. Tirzepatide would likely benefit glycaemic control and weight loss for those living with either prediabetes or T2D and obesity. These outcomes would be important to determine when evaluating the benefit–cost of these medicines in such a high-risk patient group. Indeed, the management of T2D has transformed over the past 15–20 years, as the focus of diabetes treatments has shifted towards a more generalized view of patient outcomes, particularly regarding MACE, following guidance issued by the Food and Drug Administration in 2008. A similar shift in the focus of management of people with MASLD would likely have a major impact on their clinical care and outcomes too.^105^

This is an area of current research interest, and numerous novel biomarkers have been reported to assess cardiovascular or renal risk. Cardiovascular biomarkers, such as apolipoprotein B and the apolipoprotein B:A ratio, high-sensitivity C-reactive protein, N-terminal pro-B-type natriuretic peptide and interleukin-6, amongst others, are under investigation.^106^ There are also novel biomarkers under investigation for nephropathy, which have been recently reviewed elsewhere.^107^ However, dedicated studies validating these biomarkers in patients with MASLD would be useful. With the growing number of repurposed therapies for T2D, there is a major benefit of identifying such overlapping conditions to focus therapy. For example, the use of glucagon-l ike peptide-1 receptor analogues or sodium-glucose co-transporter-2 inhibitors is associated with numerous relevant health benefits in people with T2D, such as preserving renal function, improving cardiovascular and heart failure outcomes and improving measures of steatohepatitis.^108^

Finally, a review of the issue of access to various blood-based biomarker tests at individual national or even local levels with the key stakeholders is needed to implement any strategy, for example, the place in which screening would take place. Indeed, the prospective studies to date evaluating a screening process have taken place in the community setting, secondary care services or a combination of both.^42,97,98^ This may be further complicated in real-l ife practice, as reliable access to second-l ine tests, such as the ELF test or TE, is required, and these are not universally available. As such, the development of strategies to improve access to cost-effective biomarkers for MASLD fibrosis or the development of newer biomarkers for MASLD fibrosis, which are both accessible and cost effective, is paramount to implementing MASLD screening in practice.

## Conclusions

Undeniably, MASLD is an important disease that has gained a lot of both research and clinical interest in the last 20 years. From this interest, numerous non-patented and patented non-i nvasive tests have been developed to help identify advanced fibrosis in people with MASLD.

Clearly, there remain concerns around the use of these tests, but they do appear to have sufficient accuracy and may be of clinical benefit when used in combination to permit MASLD screening in people with T2D. Further work to apply these screening algorithms in prospective studies would facilitate updated cost analyses and discussions accounting for recent therapeutic advances. Clinician consideration of the cardiorenal complications of T2D and MASLD is important, and investigating the practical use of simultaneous biomarker testing for cardiovascular, renal and hepatic comorbidities in T2D is of major interest.

Ultimately, the goal should be to translate the therapeutic benefits of novel agents at the earliest stage of the disease to help this patient group, which requires a screening process. Historically, this has been a difficult disease state to treat, but the benefit in this area has the potential for major reductions in the cardiovascular and liver-related morbidity and mortality frequently seen in this ever-growing number of people.
